# Prognostic Impact of Bone Mineral Density Reduction During Neoadjuvant Chemotherapy (NAC) in Patients Undergoing NAC Followed by Esophagectomy for Esophageal Cancer

**DOI:** 10.1002/ags3.70025

**Published:** 2025-04-16

**Authors:** Kazuhide Sato, Keita Takahashi, Yoshitaka Ishikawa, Naoko Fukushima, Takahiro Masuda, Takanori Kurogochi, Masami Yuda, Akira Matsumoto, Kazuto Tsuboi, Yuichiro Tanishima, Fumiaki Yano, Ken Eto

**Affiliations:** ^1^ Division of Gastrointestinal Surgery, Department of Surgery Jikei University School of Medicine Tokyo Japan

**Keywords:** bone mineral density reduction, esophageal cancer, neoadjuvant chemotherapy

## Abstract

**Background:**

Our previous study suggested that low bone mineral density (BMD), known as osteopenia, was a poor prognostic factor in patients who underwent esophagectomy for esophageal cancer (EC).

Meanwhile, the association between BMD reduction during neoadjuvant chemotherapy (NAC) and the worse prognosis remains unknown, although esophagectomy after NAC is the first option for the treatment of advanced esophageal squamous cell carcinoma (ESCC). Therefore, this study intended to investigate the prognostic impact of BMD reduction during NAC.

**Method:**

A total of 101 ESCC patients who underwent curative Mckeown esophagectomy after NAC between January 2008 and December 2019 were analyzed. BMD reduction (+) was defined as ≥ 6.8% of the BMD reduction rate during NAC. The patients were classified into the BMD reduction (+) group (*n* = 32) and the BMD reduction (−) group (*n* = 69) by measuring the BMD reduction during NAC.

**Results:**

Overall survival (OS) and relapse‐free survival (RFS) in the BMD reduction (+) group were significantly worse than those in the BMD reduction (−) group (*p* < 0.01). In multivariate analysis, BMD reduction was associated with worse OS (Hazard ratio [HR], 2.95; 95% confidence interval [CI], 1.44–6.05) and RFS (HR, 2.29; 95% CI, 1.30–4.03). Moreover, low skeletal muscle index before NAC was an independent risk factor for BMD massive reduction (Odds ratio, 6.21; 95% CI, 2.10–18.30).

**Conclusions:**

BMD reduction during NAC was considered to be an adverse prognostic factor for OS and RFS in patients underwent NAC followed by esophagectomy for ESCC.

## Graphical abstract

The association between BMD reduction during NAC and the worse prognosis remains unknown, although NAC followed by esophagectomy is a standard treatment for advanced ESCC. This study appeared that BMD massive reduction was an independent prognostic factor on OS (HR, 3.30; 95% confidence interval [CI], 1.69–6.55) and RFS (HR, 2.08; 95% CI, 1.19–3.63).

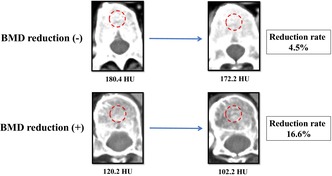

## 1 Introduction

Esophageal cancer (EC) is one of the major digestive tract malignancies worldwide, and in Japan, esophageal squamous cell carcinoma (ESCC) is known as the predominant histological type [1]. In recent years, the treatment of EC has undergone remarkable changes. Based on the results of the JCOG9907 trial and the subsequent JCOG1109 trial, neoadjuvant cisplatin and 5‐fluorouracil (5‐FU) (CF) therapy or docetaxel, cisplatin, and 5‐FU (DCF) therapy followed by esophagectomy has been established as the standard treatment for the patients with advanced ESCC or ESCC with lymph node metastasis [2, 3]. Meanwhile, esophagectomy and the perioperative treatment are reported to be highly invasive procedure with high morbidities and mortalities [4, 5]. Recent studies reported that preoperative frailty in elderly EC patients could impact postoperative complications and prognosis [6, 7]. Therefore, Continuous frailty assessment is considered to be crucial.

Osteopenia and sarcopenia are one of the components of frailty, and key factors causing falls, and fractures in older people [8]. These changes are considered affected by genetic, nutritional, hormonal factors, and life style [9, 10, 11, 12]. Sarcopenia, which was evaluated as low skeletal muscle index (SMI), was reported to cause poor survival in patients with EC [13, 14]. Furthermore, the reduction of skeletal muscle volume during neoadjuvant chemotherapy (NAC) was a prognostic risk factor in patients undergoing esophagectomy for EC [15, 16]. Meanwhile, osteopenia was defined as a low bone mineral density (BMD) assessed by calculating the average Hounsfield unit (HU) of the 11th thoracic vertebral core (Th11) on computed tomography (CT) [17]. Furthermore, previous studies suggested that preoperative osteopenia was associated with worse prognosis in patients with colorectal and biliary cancers [18, 19].

Regarding EC, our previous report suggested that preoperative osteopenia correlated with a worse prognosis after esophagectomy [7]. Meanwhile, the association between BMD change during NAC and a poor prognosis after esophagectomy in ESCC patients remains unclear. Thus, this study aimed to elucidate the impact of BMD massive reduction on OS and RFS in ESCC patients who underwent esophagectomy after NAC.

## 2 Materials and Methods

### 2.1 Patients

A total of 112 ESCC patients who received Mckeown esophagectomy after NAC in our institute from January 2008 to December 2019 were enrolled. Of those, 11 patients who underwent non‐curative resection were excluded. Clinical and pathological data, intra and postoperative status, prognosis including overall survival (OS), relapse‐free survival (RFS) were obtained from our database. The tumor clinical and pathological stage was complied with the 8th TNM classification of the Union for International Cancer Control [20].

### 2.2 Treatment Strategies for ESCC

Our ESCC treatment strategy has complied with the treatment guideline by the Japan Esophageal Society [21]. cT1 ESCC without lymph node metastasis cases received upfront Mckeown esophagectomy or definitive chemoradiotherapy (dCRT). cT2‐3 ESCC or lymph node metastasis positive cases underwent Mckeown esophagectomy after NAC. cStage IVa (T4 tumor) cases or those who refused surgery in spite of the stage, definitive chemoradiotherapy (dCRT) was implemented. Salvage esophagectomy was indicated if EC remains or develops recurrence after dCRT. As NAC regimens, 2 cycles of CF or 2 cycles of DCF were implemented 4 weeks per cycle. Pathological response to NAC was defined as five categories according to the Response Evaluation Criteria in Solid Tumors classification [22]. Adverse effects and grades during NAC were expressed according to the National Cancer Institute Common Terminology Criteria for Adverse Events (version 4.0).

### 2.3 Definitions of BMD Before and After NAC, and BMD Reduction

**FIGURE 1:**
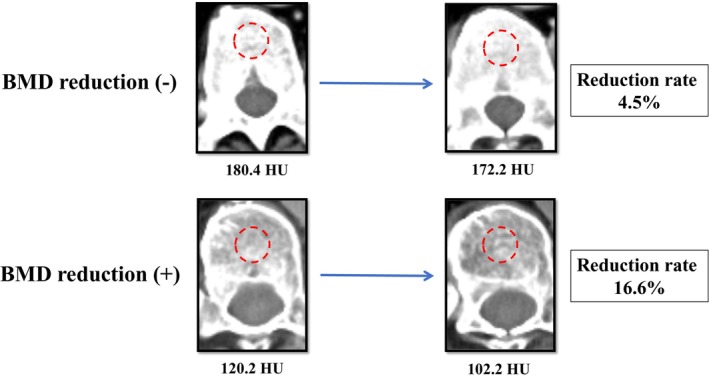
Measurement of bone mineral density (BMD) on the trabecular bone, with calculation of the average pixel density within a circle in the midvertebral core at the bottom of the 11th thoracic vertebral level.

BMD before and after NAC were measured using enhanced CT. BMD value was defined as the average HU of the midvertebral area at the lower level of the 11th thoracic vertebra on CT (Figure 1). The BMD reduction rate was calculated as follows: (BMD before NAC—BMD after NAC)/BMD before NAC × 100 (%).

### 2.4 Definitions of SMI Before and After NAC and SMI Reduction

SMI was also measured using enhanced CT before and after NAC. Synapse VINCENT image analysis system (Fujifilm Medical, Tokyo, Japan) was used to calculate SMI. First, total skeletal muscle volume (cm^2^) was measured at the cross‐sectional area of the lower level of L3 (Figure S1). After that, SMI (cm^2^/m^2^) was calculated using the following formula: total skeletal muscle volume (L3)/height (m)^2^. SMI reduction rate was calculated as follows: (SMI before NAC—SMI after NAC)/SMI before NAC × 100 (%).

### 2.5 Statistical Analysis

All data were statistically analyzed using EZR [23]. Data were shown as number (%) or the median (interquartile range). OS and RFS were evaluated using the Kaplan–Meier method and the log‐rank test for the statistical analysis. Adverse prognostic factors on OS and RFS were detected by Cox proportional hazards model. Regarding continuous variables in survival analyses, these cutoff values was determined using a time‐dependent receiver operating characteristic curve (ROC) with mortality at 5 years postoperatively as the outcome, and then BMD reduction (+) was defined as ≥ 6.8% of the BMD reduction rate. Meanwhile, continuous variables in risk analysis for the BMD massive reduction was determined using ROC curve with the BMD reduction rate ≥ 6.8% as the outcome. Statistically significant was defined as A probability level of below 0.05.

## 3 Results

### 3.1 Patient Backgrounds

Patients were initially categorized into the BMD reduction (—) (*n* = 69) and the BMD reduction group (+) (*n* = 32). The prevalence of neoadjuvant therapy and cT were significantly different between the groups (Table 1).

**TABLE 1:** Preoperative characteristics.

Variables	BMD reduction (−) *n* = 69	BMD reduction (+) *n* = 32	*p*
Age (years)	66.0 (62.0, 71.0)	65.5 (59.0, 71.0)	0.47
Gender			
Male	63 (91.3)	25 (78.1)	0.11
Female	6 (8.7)	7 (21.9)	
ASA‐PS			
1	17 (24.6)	8 (25.0)	0.46
2	52 (75.4)	23 (71.9)	
3	0 (0.0)	1 (3.1)	
Neoadjuvant chemotherapy			
Docetaxel+Cisplatin+5‐FU	67 (97.1)	28 (87.5)	0.08
Cisplatin+5‐FU (CF)	2 (2.9)	4 (12.5)	
Tumor location			
Upper third	12 (16.4)	6 (15.4)	0.56
Middle third	33 (45.2)	14 (35.9)	
Lower third	28 (38.4)	19 (48.7)	
*Clinical tumor stage (UICC 8th)*			
Depth of tumor			
cT1	7 (10.1)	5 (15.6)	0.01
cT2	18 (26.1)	1 (3.1)	
cT3	44 (63.8)	26 (81.2)	
Lymph node metastasis			
cN0	16 (23.2)	9 (28.1)	0.54
cN1	37 (53.6)	13 (40.6)	
cN2	15 (21.7)	9 (28.1)	
cN3	1 (1.4)	1 (3.1)	
Clinical stage			
cStageI	3 (6.1)	4 (16.0)	0.77
cStageII	17 (34.7)	7 (28.0)	
cStageIII	21 (42.9)	10 (40.0)	
cStageIV	3 (6.1)	1 (4.0)	

*Note:* Data expressed as number (%) or Median (Interquartile range).

Abbreviations: ASA‐PS, American Society of Anestheologists‐physical status; BMD, bone mineral density; BMI, body mass index; FU, fluorouracil; UICC, the Union for International Cancer Control.

### 3.2 Differences in Patients' Serum Albumin or Body Composition

Body mass index (BMI) before NAC was significantly lower in the BMD reduction group (+) than in the other group, while BMI after NAC was comparable between the two groups. BMI reduction rate was significantly higher in the BMD reduction group (+) than in the other group. Other characteristics were not significantly different between the two groups. BMD after NAC was significantly lower in the BMD reduction group (+) than in the BMD reduction (—) group (*p* < 0.01). Meanwhile, SMI before NAC and the reduction rate were significantly lower in the BMD reduction group (+) than in the BMD reduction (—) group (*p* = 0.02, *p* = 0.01, respectively) (Table 2).

**TABLE 2:** Changes of body composition and adverse effects during NAC.

Variables	BMD reduction (−) *n* = 69	BMD reduction (+) *n* = 32	*p*
BMI (kg/m^2^) before NAC	21.9 (20.4, 23.4)	20.9 (18.2, 22.5)	0.03
BMI after NAC (kg/m^2^)	21.3 (19.7, 23.1)	20.1 (18.4, 22.5)	0.09
BMI reduction rate (%)	−1.59 (−5.47–0.67)	1.42 (−3.88–7.64)	0.046
Serum Albumin			
Before NAC (g/dL)	4.10 (3.90, 4.40)	4.05 (3.80, 4.32)	0.46
After NAC (g/dL)	3.80 (3.60, 4.00)	3.80 (3.68, 4.03)	0.55
Reduction rate during NAC (%)	7.1 (2.3, 14.3)	5.59 (1.06, 13.4)	0.35
BMD			
Before NAC (HU)	141 (118, 171)	154 (127, 198)	0.19
After NAC (HU)	154 (127, 185)	128 (95, 148)	< 0.01
Reduction rate during NAC (%)	−5.2 (−15.0, 0.8)	18.0 (12.0, 33.0)	< 0.01
Skeletal muscle index			
Before NAC (cm^2^/m^2^)	52.0 (48.0, 56.0)	49.0 (44.0, 54.0)	0.02
After NAC (cm^2^/m^2^)	50.1 (46.3, 54.5)	49.3 (46.5, 53.7)	0.5
Reduction rate during NAC (%)	2.86 (−0.1, 8.0)	−1.37 (−7.80, 4.13)	0.01
Adverse effects during NAC			
Neutropenia (Any grade)	65 (94.2)	25 (78.1)	0.03
Neutropenia (Grade 3 or more)	55 (79.7)	18 (56.2)	0.02
Lymphopenia (Any grade)	8 (11.6)	5 (15.6)	0.54
Elevated AST (Any grade)	0	2 (6.2)	0.11
Elevated ALT (Any grade)	3 (4.3)	1 (3.1)	1
Elevated creatine (Any grade)	9 (13.0)	1 (3.1)	0.16

Abbreviations: BMD, bone mineral density; BMI, body mass index; HU, Hounsfield unit; NAC, neoadjuvant chemotherapy.

### 3.3 Intra‐ and Postoperative Outcomes

Intra‐ and postoperative outcomes were comparable between the two groups. Additionally, pathological tumor stage or pathological response to NAC was not significantly different (Table 3).

**TABLE 3:** Intra‐and postoperative characteristics.

Variables	BMD reduction (−) *n* = 69	BMD reduction (+) *n* = 32	*p*
Type of thoracic surgery			
Thoracoscopic	46 (66.7)	17 (53.1)	0.29
Thoracotomy	22 (31.9)	15 (46.9)	
Transhiatal	1 (1.4)	0 (0.0)	
Reconstruction route			
Retrosternal	61 (88.4)	28 (87.5)	0.43
Posterior mediastinal	8 (11.6)	3 (9.4)	
Antethoracic	0 (0.0)	1 (3.1)	
Operation time (min)	556 (494, 603)	554 (542, 570)	0.75
Blood loss (ml)	310 (200, 610)	348 (141, 595)	0.89
Postoperative complications			
Anastomotic leakage	6 (8.7)	7 (21.9)	0.11
Pneumonia	14 (20.3)	5 (15.6)	0.79
Recurrent laryngeal nerve palsy	23 (33.3)	5 (15.6)	0.09
Length of hospital stay (days)	21 (17, 33)	21 (18, 52)	0.43
*Pathologic tumor stage* (*UICC 8th*)			
Depth of tumor			
ypT0‐1	28 (40.6)	12 (37.5)	0.98
ypT2	11 (15.9)	5 (15.6)	
ypT3	28 (40.6)	14 (43.8)	
ypT4	2 (2.9)	1 (3.1)	
Lymph node metastasis			
ypN0	26 (37.7)	13 (40.6)	0.22
ypN1	21 (30.4)	5 (15.6)	
ypN2	14 (20.3)	6 (18.8)	
ypN3	8 (11.6)	8 (25.0)	
Pathologic stage			
ypStage0‐I	13 (18.8)	8 (25.0)	0.63
ypStageII	25 (36.2)	9 (28.1)	
ypStageIII	17 (24.6)	6 (18.8)	
ypStageIV	14 (20.3)	9 (28.1)	
Pathological response to NAC			
Grade0	10 (14.5)	4 (12.5)	0.64
Grade1a	30 (43.5)	15 (46.9)	
Grade1b	11 (15.9)	8 (25.0)	
Grade2	11 (15.9)	2 (6.2)	
Grade3	7 (10.1)	3 (9.4)	

Abbreviations: BMD, bone mineral density; NAC neoadjuvant chemotherapy; UICC the Union for International Cancer Control.

### 3.4 Comparison of Overall Survival (OS) and Relapse‐Free Survival (RFS)

**FIGURE 2:**
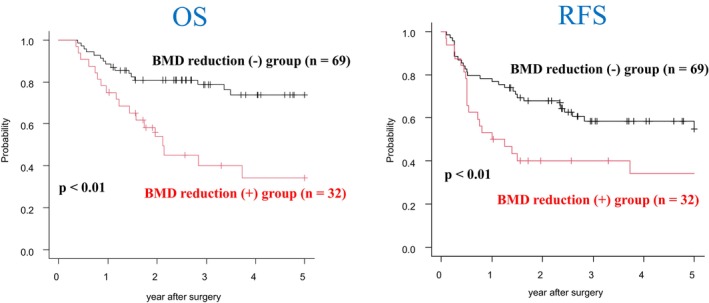
The OS and RFS between the BMD (+) group and the BMD (−) group the BMD (+) group had significantly worse OS and RFS than the BMD (−) group (*p* < 0.01, respectively).

The BMD (+) group had significantly worse OS and RFS than the BMD (—) group (*p* < 0.01, respectively) (Figure 2).

### 3.5 Impact of BMD Massive Reduction and Other Factors on OS and RFS

As shown in Table 4, the univariate analysis demonstrated that the important factors affecting a worse OS were BMI reduction rate ≥ 5.9%, BMD reduction ≥ 6.8%, ypT3‐4 and ypN positive. In the multivariate analysis, BMI reduction rate ≥ 5.9% (*p* < 0.01; HR, 2.46; 95% [CI], 1.13–5.36), SMI reduction ≥ 10.1% (*p* < 0.46; HR, 2.25; 95% [CI], 1.01–5.01), BMD reduction ≥ 6.8% (*p* < 0.01; HR, 2.95; 95% [CI], 1.44–6.05), and ypN positive (*p* < 0.01; HR, 3.69; 95% CI, 1.59–8.58) were independent adverse prognostic factors.

**TABLE 4:** Cox proportional hazard model for overall survival.

Variables	Univariate analysis HR (95% CI)	*p*	Multivariate analysis HR (95% CI)	*p*
Age ≥ 60	1.65 (0.84, 3.24)	0.15		
Gender (male)	0.69 (0.28, 1.80)	0.45		
BMI reduction rate ≥ 5.9%	3.82 (1.86, 7.87)	< 0.01	2.46 (1.13, 5.36)	< 0.01
Serum Albumin reduction ≥ 9.5%	0.72 (0.36, 1.46)	0.36		
SMI reduction ≥ 10.1%	1.88 (0.85, 4.14)	0.12	2.25 (1.01, 5.01)	0.046
BMD reduction ≥ 6.8%	3.30 (1.69, 6.55)	< 0.01	2.95 (1.44, 6.05)	< 0.01
ASA‐PS,3	1.93 (0.26, 14.2)	0.52		
Operation time ≥ 587 min	0.71 (0.32, 1.57)	0.4		
Operative blood loss ≥ 510 ml	1.61 (0.80, 3.27)	0.32		
Postoperative complications				
Anastomotic leakage	1.43 (0.55, 3.69)	0.46		
Pneumonia	0.96 (0.40, 2.31)	0.66		
Recurrent laryngeal nerve palsy	1.12 (0.55, 2.29)	0.75		
Pathologic tumor stage				
ypT3,4	2.11 (1.07, 4.15)	0.03		
ypN positive	3.12 (1.36, 7.15)	< 0.01	3.69 (1.59, 8.58)	< 0.01
Pathological response, Grade 0–1b	2.61 (0.92, 7.41)	0.07		

Abbreviations: ASA‐PS, American Society of Anestheologists‐physical status; BMD, bone mineral density; BM,I body mass index; CI, confidence interval; HR, hazard ratio; NAC, neoadjuvant chemotherapy; SMI, skeletal muscle index.

In univariate analysis for the RFS, BMI reduction rate ≥ 5.9%, BMD reduction ≥ 6.8%, ypT3‐4 and ypN positive were the significant factors. Multivariate analysis revealed that the independent factors were BMD reduction ≥ 6.8% (*p* < 0.01; HR, 2.29; 95% CI, 1.30–4.03), ypT3‐4 (*p* < 0.01; HR, 2.74; 95% CI, 1.54–4.86) and ypN positive (*p* < 0.01; HR, 2.58; 95% CI, 1.37–4.87) (Table 5).

**TABLE 5:** Cox proportional hazard model for relapse‐free survival.

Variables	Univariate analysis HR (95% CI)	*p*	Multivariate analysis HR (95% CI)	*p*
Age ≥ 60	0.71 (0.37, 1.37)	0.31		
Gender (male)	0.79 (0.36, 1.77)	0.57		
BMI reduction rate ≥ 5.9%	2.47 (1.26, 4.85)	< 0.01		
Serum Albumin reduction ≥ 9.5%	1.00 (0.97, 1.04)	0.79		
SMI reduction ≥ 10.1%	1.63 (0.81, 3.28)	0.17		
BMD reduction ≥ 6.8%	2.08 (1.19, 3.63)	0.01	2.29 (1.30, 4.03)	< 0.01
ASA‐PS,3	1.21 (0.17, 8.83)	0.85		
Operation time ≥ 587 min	1.03 (0.56, 1.88)	0.93		
Operative blood loss ≥ 510 mL	1.09 (0.62, 1.92)	0.77		
Postoperative complications				
Anastomotic leakage	1.21 (0.54, 2.69)	0.64		
Pneumonia	0.94 (0.46, 1.94)	0.87		
Recurrent laryngeal nerve palsy	1.07 (0.58, 1.95)	0.84		
Pathologic tumor stage				
ypT3,4	2.71 (1.53, 4.79)	< 0.01	2.74 (1.54–4.86)	< 0.01
ypN positive	2.56 (1.36, 4.81)	< 0.01	2.58 (1.37, 4.87)	< 0.01
Pathological response, Grade 0–1b	1.86 (0.87, 3.96)	0.11		

Abbreviations: ASA‐PS, American Society of Anestheologists‐physical status; BMD, bone mineral density; BMI, body mass index; CI, confidence interval; HR, hazard ratio; NAC, neoadjuvant chemotherapy; SMI, skeletal muscle index.

### 3.6 Risk Factors of BMD Massive Reduction

Risk factors for BMD massive reduction were investigated by a logistic regression model. The univariate analysis showed that the significant factor was SMI before NAC of < 45.5 cm^2^/m^2^ and Neutropenia during NAC. In the multivariate analysis, the independent risk factor was also SMI before NAC of < 45.5 cm^2^/m^2^ (*p* < 0.01; HR, 6.21; 95% CI, 2.10–18.30). Conversely, neutropenia during NAC was a protective factor (*p* = 0.03; HR, 0.21; 95% CI, 0.05–0.84) (Table 6).

**TABLE 6:** Logistic regression model for BMD massive reduction.

Variables	Univariate analysis OR (95% CI)	*p*	Multivariate analysis OR (95% CI)	*p*
Age ≥ 60	0.57 (0.20, 1.59)	0.28		
Gender (male)	0.34 (0.10, 1.11)	0.07		
BMI before NAC < 20.5	2.32 (0.97, 5.56)	0.06		
Albumin before NAC < 4.1 g/dL	1.46 (0.63, 3.40)	0.38		
SMI before NAC < 45.5 cm^2^/m^2^	6.06 (2.11, 17.4)	< 0.01	6.21 (2.10, 18.30)	< 0.01
BMD before NAC < 161 HU	0.68 (0.29, 1.58)	0.37		
Tumor stage				
cT3, 4	2.46 (0.89, 6.79)	0.08		
cN positive	0.77 (0.30, 2.00)	0.59		
DCF regimen	0.21 (0.04, 1.21)	0.08		
Neutropenia (Grade 3 or more)	0.33 (0.13–0.82)	0.02	0.21 (0.05–0.84)	0.03

Abbreviations: OR, odds ratio; CI, confidence interval; NAC, neoadjuvant chemotherapy; SMI, skeletal muscle index; BMD, bone mineral density; HU, Hounsfield unit; DCF, Docetaxel+Cisplatin+5‐FU.

## 4 Discussion

To our knowledge, this study is the first to demonstrate that BMD reduction during NAC for ESCC was associated with worse prognosis after esophagectomy. Additionally, the risk factor for BMD reduction during NAC was a pretherapeutic SMI of < 45.5 cm^2^/m^2^, suggesting that nutrition support and rehabilitation during NAC might improve BMD reduction and the subsequent prognosis.

A possible explanation for BMD massive reduction during NAC and worse prognosis after esophagectomy is that cancer derived proinflammatory cytokines including Interleukin (IL)‐1, IL‐6, and tumor necrosis factor (TNF)‐α, may activate NF‐κβ and c‐Fos, resulting in the differentiation of osteoclast genesis in patients with cancer. Yokota et al. reported that the stimulation of human CD14+ monocyte with TNF‐α and IL‐6 can trigger the differentiation of osteoclast‐like cells [24]. Additionally, the receptor activator of NF‐κβ ligand released by cancer cells may contribute to the activation of osteoclastogenesis mediated through inhibitory kappa kinases and the NF‐κβ pathway [25]. These proinflammatory cytokines may elicit cancer progression and BMD reduction in patients with cancer. Another possible mechanism is that BMD massive reduction may contribute to intolerance to treatment for postoperative recurrence as well as a decline in postoperative ADL, resulting in worse prognosis after esophagectomy.

Regarding the association between frailty progression and worse prognosis after esophagectomy, previous studies have demonstrated that SMI reduction during NAC or neoadjuvant chemoradiation therapy was associated a worse prognosis after esophagectomy [26, 27]. Our additional analysis showed that BMD and SMI were respectively correlated each other before and after NAC (Figure S2). Also, this study revealed that low SMI before NAC was as a risk factor for BMD massive reduction. Low SMI and low BMD are considered key factors of the aging process, causing fractures, and frailty in older people. Thus, BMD reduction may also be considered as important as SMI reduction during NAC in ESCC patients. Meanwhile, neutropenia during NAC was a protective factor for BMD massive reduction. This possible explanation is that more patients in BMD massive reduction group underwent CF therapy than the patients in the other group, although the pathophysiological mechanism remains unknown.

The finding that BMD massive reduction was a poor prognostic factor is considered more valuable than the previous study, which identified preoperative osteopenia as a poor prognostic factor, because there is potential for intervention to mitigate frailty progression during NAC. As a preventive measure, we hypothesized that earlier rehabilitation and vitamin D supplementation during NAC may improve the frailty after NAC. Halliday et al. reported that early rehabilitation during NAC in EC patients decreased SMI reduction. Considering the correlation BMD and SMI values before and after NAC described in Figure S2, rehabilitation during NAC may help prevent BMD reduction. Meanwhile, vitamin D supplementation may be another solution to suppress BMD reduction. Yamada et al. suggested that chemoradiation therapy decreased the BMD in patients with low serum vitamin D levels. Therefore, supplementation of vitamin D may prevent BMD reduction, resulting in the improvement of the prognosis subsequently. Furthermore, 1,25(OH)2D suppresses the activation of proinflammatory factors, such as IL‐1 and IL‐6 [28, 29], suggesting that Vitamin D itself may possess potential anti‐tumor effects.

This study includes some limitations. First, this is a retrospective cohort study investigated in a single institution, resulting in small population. Second, the cutoff value of osteopenia and sarcopenia differs from the studies, because the cutoff values were not generally defined. Third, the main histological type and treatment strategy for resectable EC are different between Western countries and Japan. Fourth, NAC regimens in this study were a little different from newly adopted JCOG 1109 regimen. Therefore, there is a possibility that this study's results may not apply to the patients treated with the JCOG 1109 regimen.

## 5 Conclusion

BMD reduction during NAC was considered to be an adverse prognostic factor for OS and RFS in patients underwent esophagectomy after NAC for ESCC.

## Author Contributions

**Kazuhide Sato:** conceptualization, data curation, investigation, writing – original draft. **Keita Takahashi:** conceptualization, data curation, investigation, visualization, writing – review and editing. **Yoshitaka Ishikawa:** data curation, formal analysis. **Naoko Fukushima:** data curation. **Takahiro Masuda:** data curation, methodology. **Takanori Kurogochi:** data curation, investigation, methodology. **Masami Yuda:** data curation, investigation, methodology. **Akira Matsumoto:** data curation, investigation, methodology. **Kazuto Tsuboi:** data curation, investigation, methodology. **Yuichiro Tanishima:** data curation, investigation, writing – review and editing. **Fumiaki Yano:** supervision. **Ken Eto:** supervision.

## Ethics Statement

The study protocol was accepted by the institutional review board of the Jikei University School of Medicine (33–303).

## Consent

The need for informed consent was waived because of the retrospective study design.

## Conflicts of Interest

The authors declare no conflicts of interest.

## Supporting information


**Figure S1.** Measurement of Skeletal Muscle index (SMI).
Total skeletal muscle volume (cm^2^) was measured at the cross‐sectional area of the lower level of L3. SMI (cm^2^/m^2^) was calculated using the following formula: total skeletal muscle volume/height (m)^2^.
**Figure S2.** Correlation between BMD and SMI before and after NAC. BMD and SMI were correlated each other before and after NAC (*p* = 0.02, respectively).
