# Supplementary material for: Prognostic Impact of Bone Mineral Density Reduction During Neoadjuvant Chemotherapy (NAC) in Patients Undergoing NAC Followed by Esophagectomy for Esophageal Cancer
Source: Ann Gastroenterol Surg. 2025 Apr 16;9(5):933–41. doi: 10.1002/ags3.70025 (PMC12414600; doi:10.1002/ags3.70025)
Supplement: Supplementary file 1 — Figure S1. Measurement of Skeletal Muscle index (SMI). Total skeletal muscle volume (cm2) was measured at the cross‐sectional area of the lower level of L3. SMI (cm2/m2) was calculated using the following formula: total skeletal muscle volume/height (m)2. Figure S2. Correlation between BMD and SMI before and after NAC. BMD and SMI were correlated each other before and after NAC (p = 0.02, respectively). [file AGS3-9-933-s001.pptx]

## Slide 1
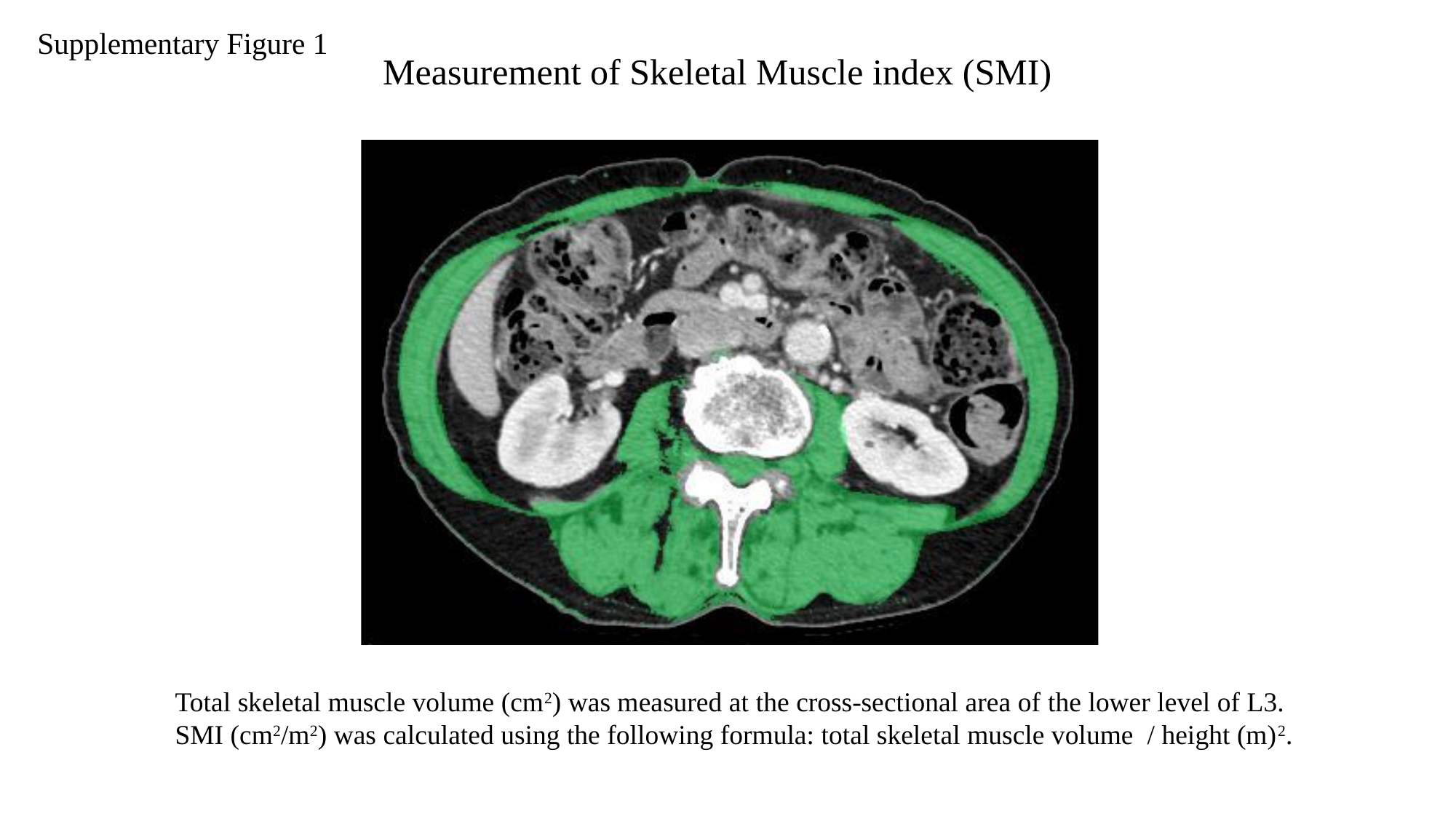

Supplementary Figure 1
Measurement of Skeletal Muscle index (SMI)
Total skeletal muscle volume (cm2) was measured at the cross-sectional area of the lower level of L3.
SMI (cm2/m2) was calculated using the following formula: total skeletal muscle volume / height (m)2.

## Slide 2
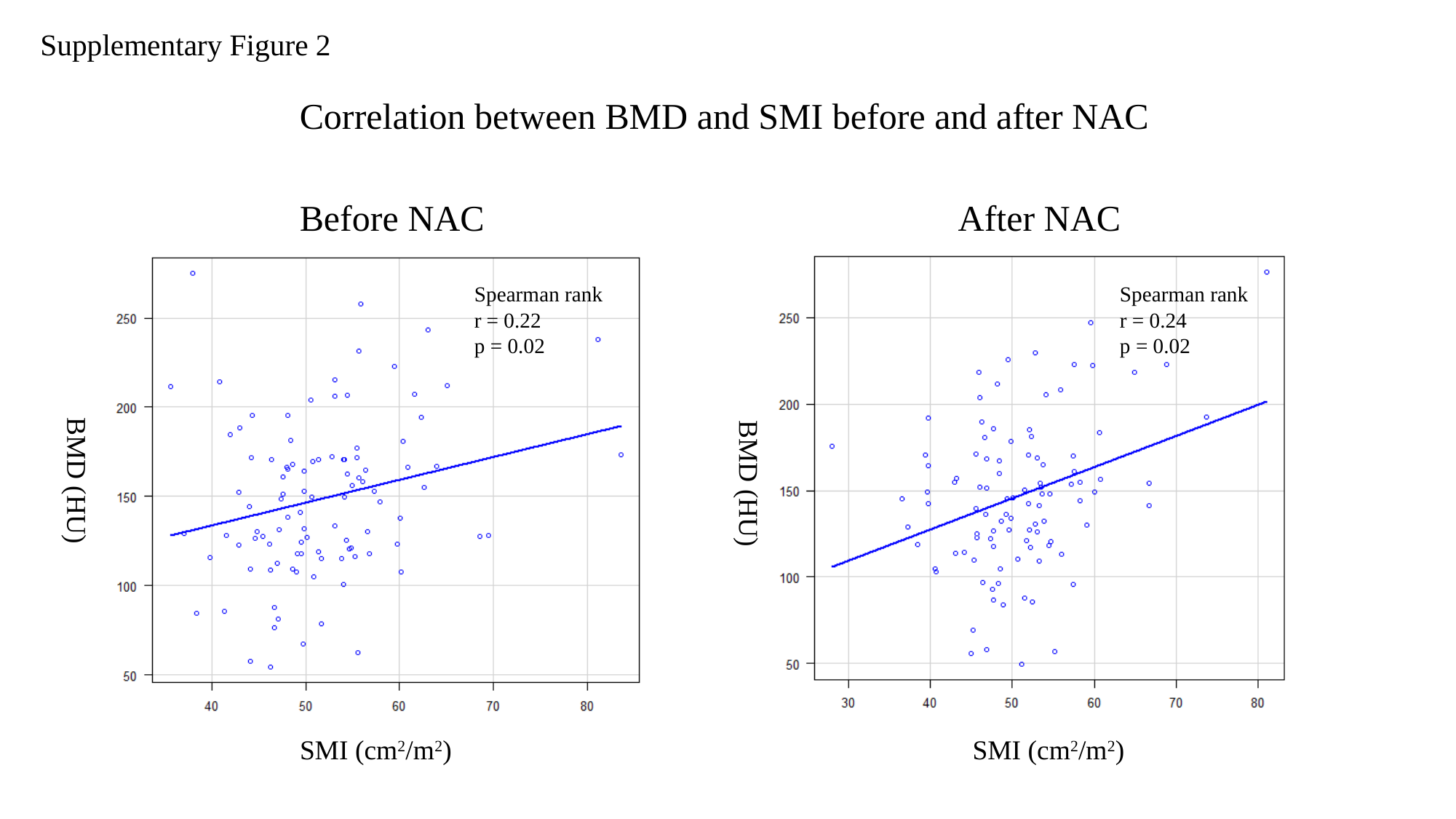

Supplementary Figure 2
Correlation between BMD and SMI before and after NAC
After NAC
Before NAC
Spearman rank
r = 0.22
p = 0.02
Spearman rank
r = 0.24
p = 0.02
BMD (HU)
BMD (HU)
SMI (cm2/m2)
SMI (cm2/m2)
